# Two New Rapid SNP-Typing Methods for Classifying *Mycobacterium tuberculosis* Complex into the Main Phylogenetic Lineages

**DOI:** 10.1371/journal.pone.0041253

**Published:** 2012-07-20

**Authors:** David Stucki, Bijaya Malla, Simon Hostettler, Thembela Huna, Julia Feldmann, Dorothy Yeboah-Manu, Sonia Borrell, Lukas Fenner, Iñaki Comas, Mireia Coscollà, Sebastien Gagneux

**Affiliations:** 1 Swiss Tropical and Public Health Institute, Basel, Switzerland; 2 University of Basel, Basel, Switzerland; 3 Division of Mycobacterial Research, Medical Research Council, National Institute for Medical Research, London, United Kingdom; 4 Noguchi Memorial Institute for Medical Research, University of Ghana, Legon, Ghana; 5 Institute of Social and Preventive Medicine, University of Bern, Bern, Switzerland; 6 Genomics and Health Unit, Centre for Public Health Research, Valencia, Spain; 7 CIBER Epidemiologìa y Salud Pública, Madrid, Spain; St. Petersburg Pasteur Institute, Russian Federation

## Abstract

There is increasing evidence that strain variation in *Mycobacterium tuberculosis* complex (MTBC) might influence the outcome of tuberculosis infection and disease. To assess genotype-phenotype associations, phylogenetically robust molecular markers and appropriate genotyping tools are required. Most current genotyping methods for MTBC are based on mobile or repetitive DNA elements. Because these elements are prone to convergent evolution, the corresponding genotyping techniques are suboptimal for phylogenetic studies and strain classification. By contrast, single nucleotide polymorphisms (SNP) are ideal markers for classifying MTBC into phylogenetic lineages, as they exhibit very low degrees of homoplasy. In this study, we developed two complementary SNP-based genotyping methods to classify strains into the six main human-associated lineages of MTBC, the “Beijing” sublineage, and the clade comprising *Mycobacterium bovis* and *Mycobacterium caprae*. Phylogenetically informative SNPs were obtained from 22 MTBC whole-genome sequences. The first assay, referred to as MOL-PCR, is a ligation-dependent PCR with signal detection by fluorescent microspheres and a Luminex flow cytometer, which simultaneously interrogates eight SNPs. The second assay is based on six individual TaqMan real-time PCR assays for singleplex SNP-typing. We compared MOL-PCR and TaqMan results in two panels of clinical MTBC isolates. Both methods agreed fully when assigning 36 well-characterized strains into the main phylogenetic lineages. The sensitivity in allele-calling was 98.6% and 98.8% for MOL-PCR and TaqMan, respectively. Typing of an additional panel of 78 unknown clinical isolates revealed 99.2% and 100% sensitivity in allele-calling, respectively, and 100% agreement in lineage assignment between both methods. While MOL-PCR and TaqMan are both highly sensitive and specific, MOL-PCR is ideal for classification of isolates with no previous information, whereas TaqMan is faster for confirmation. Furthermore, both methods are rapid, flexible and comparably inexpensive.

## Introduction

Genotyping of human-associated *Mycobacterium tuberculosis* complex (MTBC) plays an increasing role for understanding the epidemiology and biology of tuberculosis (TB) [1], [2]. On the one hand, genotyping techniques are key for standard molecular epidemiological investigations of TB such as defining chains of ongoing transmission and differentiating patient relapse from exogenous re-infection [1]. On the other hand, strain genotyping in MTBC is important for defining the evolutionary background of clinical isolates. There is mounting evidence that the strain genetic background might influence the outcome of TB infection and disease. Experimental studies have shown that clinical strains differ in immunogenicity and virulence, but whether this variation translates into comparable clinical phenotypes is unclear [2]–[9]. To study the effect of strain variation and detect relevant genotype-phenotype associations, suitable phylogenetic markers and appropriate genotyping methods are required [10].

Several methods for genotyping of MTBC have been developed over the past years [11]. Two of the most popular methods, spoligotyping [12] and MIRU-VNTR [13], rely on repetitive DNA elements, and are currently considered the gold standard for TB transmission studies [1], [13]. However, due to their propensity for convergent evolution and resulting homoplasies [10], inferring robust phylogenies using these methods can be problematic. Moreover, because of these homoplasies, classification of MTBC strains into robust phylogenetic lineages is not always possible and misclassification can occur [14], [15]. In addition to mobile genetic elements (e.g. IS*6110* [16]), repetitive DNA (DR region [17], MIRU-VNTR [18]), and large sequence polymorphisms [19], [20], single nucleotide polymorphisms (SNPs) have become available as phylogenetic markers for MTBC. SNPs are ideal markers for genotyping of MTBC, as they represent unique events and show virtually no homoplasy [10]. Several studies have reported the use of SNPs for phylogenetic classification of MTBC [21]–[29]. However, no consensus has yet been reached as to what set of SNPs should be used for standard phylotyping of MTBC. Importantly, not all phylogenetically informative SNPs will be equally amendable to all possible SNP-typing platforms [30], [31]. In MTBC, some of the methods that have been proposed are allele-specific PCR [32], real-time PCR [33], Sanger sequencing [34], SNaPshot minisequencing [28], iPlex Gold (SEQUENOM Inc.) [29] and MLPA [25]. All of these methods differ in their technical requirements. Hence, depending on the particular technique used, different, but phylogenetically equivalent SNP sets will be required. In addition, these typing methods vary largely in their throughput, cost, and flexibility. With respect to the latter, we identified a lack of methods, which are at the same time flexible and rapid (<1 day/96 samples), and amendable to a variable number of strains and a limited number of SNPs (5–50).

**Figure 1:**
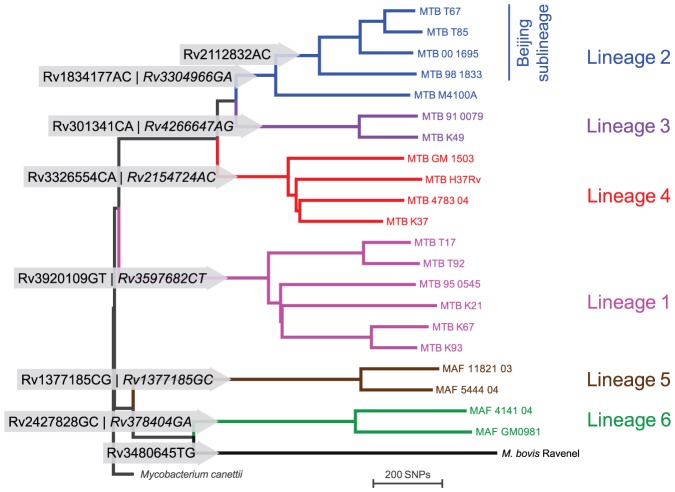
Phylogenetic tree of 22 whole genome sequences of MTBC plus *Mycobacterium canettii* as outgroup, and canonical SNPs used for MOL-PCR and TaqMan assays. Rv-numbers in grey arrows represent SNPs used for MOL-PCR and TaqMan in this study, respectively, and nucleotide change at the position in the annotated reference sequence of H37Rv [56]. The first number indicates the SNP used for MOL-PCR, and the italic number the SNP used for TaqMan. Assays for the “Beijing” sublineage of Lineage 2 and for *M. bovis*/*M. caprae* were developed only for MOL-PCR (modified from [51]).

Recently, whole genome sequences of a global collection of 21 MTBC strains representing the six main lineages of human-associated MTBC became available [35]. The 9,037 SNPs identified in these genomes represent an ideal starting point for extracting diagnostic or “canonical” SNPs, which are lineage-specific, to design phylogenetically robust genotyping assays [36]. In this study, we present two new and complementary SNP-typing methods for MTBC. These methods are based on two phylogenetically equivalent sets of SNP markers that are specific for the 6 main human-associated lineages of MTBC. Additionally, we present SNPs specific for the clade comprising *Mycobacterium bovis* and *Mycobacterium caprae* [26], and for the “Beijing”-sublineage of Lineage 2 (East-Asian) (Figure 1). “Beijing” strains are of special epidemiological interest because of their repeated association with drug resistance and hypervirulence in infection models [37], [38]. The *M. bovis*/*M. caprae* specific SNP does not capture the other animal-associated strains, i.e. *M. pinnipedii*, *M. microti*, *M. orygis*, *M. mungi* and the Dassie bacillus.

**Figure 2:**
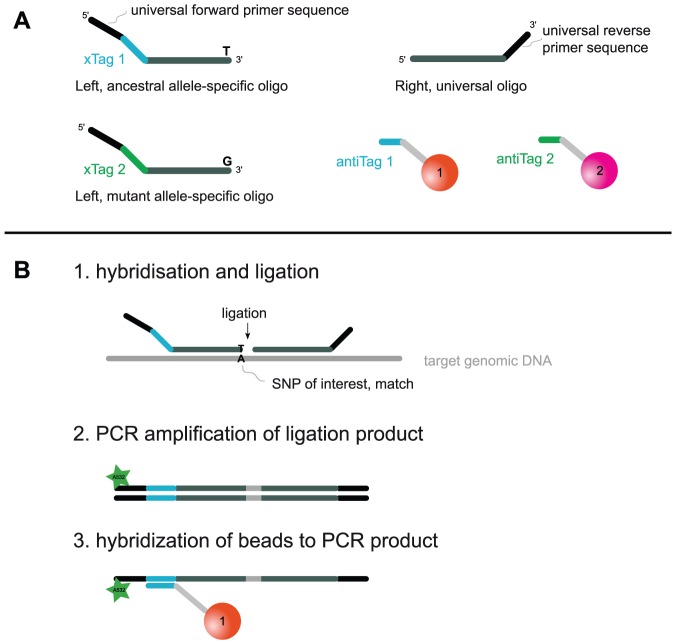
Schematic illustration of MOL-PCR. A. Three oligonucleotides and two fluorescently labelled beads are used for interrogation of one SNP. B. MOL-PCR consists of three steps. 1. One of two competitive allele-specific left-hand probe oligonucleotides (LPO) and one universal right-hand probe oligonucleotide (RPO) are hybridized to the template DNA and ligated. 2. With PCR and a reporter-labelled forward primer, the ligated oligonucleotides (LPO+RPO) are amplified. 3. After denaturation of the PCR product, allele-specific fluorescent beads carrying an allele-specific *antiTag* sequence are hybridized to the amplicons. This will result in beads carrying reporter fluorescence (bead 1 in example) and beads not carrying fluorescence (bead 2). Reporter fluorescence (Alexa532) per bead is measured with a flow cytometric device (Luminex). For the 8-plex assay, a total of 24 oligonucleotides and 16 beads are used in the same reaction.

The first SNP-typing method is MOL-PCR (multiplexed oligonucleotide ligation PCR) [39], which uses allele-specific ligation for allele discrimination, singleplex PCR for signal amplification and fluorescent microspheres (beads) for the signal detection on a flow cytometer (Figure 2, see Methods). A Luminex device is needed; i.e. a flow cytometric platform for various nucleic acid and immunological assays [40]–[42], which allows for simultaneous interrogation of 50 biallelic SNPs. MOL-PCR is flexible for both the number of SNPs as well as for the number of strains tested (individual tubes to 96-well plates). We developed an 8-plex SNP-typing assay for the identification of the main phylogenetic lineages of MTBC. As a second method, we present SNP-typing with TaqMan real-time PCR. As a commercially available system, TaqMan has proved to be sensitive and specific in numerous studies for various applications and species [43]–[46], and is therefore considered the standard for SNP-typing in our laboratory [14], [20]. Reactions are performed in a 96-well format in less than 2 hours. In contrast to MOL-PCR, only one SNP is assessed per reaction.

## Methods

### Ethics statement

All MTBC strains used in this study were from published reference collections [14], [20], [26], [47]–[49] or from an ongoing molecular epidemiology study on tuberculosis in Nepal. This study was ethically approved by the Nepal Health Research Council (NHRC), Kathmandu, Nepal, and the Ethics Committee of Basel, Switzerland (EKBB). Written informed consent was obtained from all Nepalese patients.

### Strain panels

Two different strain panels were used in this study. A “training panel” of 46 MTBC strains was first chosen for establishing the MOL-PCR and TaqMan assays. These strains had been characterized previously by spoligotyping and were available as crude lysates and purified DNA. This training panel contained clinical strains from published reference collections [14], [20], [26], [47]–[49] and some clinical isolates from Nepal (manuscript in preparation). The training panel was composed of five to seven isolates of the 6 main phylogenetic lineage of MTBC, and additionally, *Mycobacterium bovis*, *Mycobacterium caprae*, *Mycobacterium pinnipedii* and *Mycobacterium microti* as controls for the *M. bovis*/*M. caprae-specific* SNP in the MOL-PCR assay. For each of these strains, SNP-typing was done by MOL-PCR and TaqMan for both crude lysates and purified DNA. Allele calls, i.e. lineage assignments, were obtained for all data points, and results between the two methods compared. As it was not possible to use the same SNPs for both MOL-PCR and TaqMan (due to probe design restrictions), we compared the lineage assignment rather than the locus-specific alleles.

A “test panel” was then chosen for the validation of MOL-PCR and TaqMan under “blind” conditions. This test panel consisted of 78 clinical MTBC isolates recently obtained from a molecular epidemiology study in Nepal. No previous genotyping had been done for these samples. The samples were cultured on Löwenstein-Jenssen (LJ) medium, frozen in tryptic soy agar and glycerol, and heat-inactivated for 1 h at 90°C. Purified DNA was available for fifteen of these strains.

### Culturing and DNA extraction

MTBC strains were grown in standard Middlebrook 7H9 medium supplemented with ADC (Becton Dickinson, Allschwil, Switzerland), Tween80 (Sigma-Aldrich) and glycerol (Sigma-Aldrich). Liquid medium and agar plates for culturing *Mycobacterium africanum* (i.e. MTBC Lineage 5 and 6) were supplemented with 40 mM sodium pyruvate (Sigma-Aldrich). All bacterial cultures were incubated at 37°C. After two weeks, 500 µL culture were heat-inactivated (1 hour at 90°C) and centrifuged. The supernatant was used as input for TaqMan and MOL-PCR. Alternatively, 100 µL of a frozen stock culture was thawed and heat-inactivated (100 µL culture added to 100 µL TE buffer, 1 hour at 90°C). From all cultures, purified genomic DNA was extracted with the CTAB method [50]. DNA concentration was determined with a Nanodrop ND-1000 device (Thermo Scientific NanoDrop Products, Wilmington, USA).

### Whole genome sequencing and SNP identification

Informative SNPs were obtained from whole genome sequencing as described before [35], [51]. In brief, short reads from Illumina-sequencing of 22 MTBC strains were mapped to the reference genome of H37Rv and strain-specific nucleotide differences were extracted as SNPs. A neighbor-joining phylogenetic tree was constructed with MEGA [52], and SNPs mapped to the tree using Mesquite [53]. Clade-specific SNPs were compiled in OpenOffice spreadsheets (openoffice.org). Position in reference to H37Rv, codon change, essentiality [54], [55], and annotated function of the gene [56] were collected for each SNP. This list was used as a starting point for oligonucleotide design. In the course of the study, 151 additional genome sequences became available (manuscript in preparation). With the total of 172 genome sequences representing the global MTBC diversity, we confirmed that the SNPs chosen were specific for all members of a lineage. In reference to the first MTBC genome sequenced and annotated [56] (Genbank AL123456.2), every SNP was named with the prefix “Rv”, followed by 7 digits that represent the position of this SNP in the genome of H37Rv, and the nucleotide change (i.e. “Rv2154724AC” for the Lineage 4-specific SNP in *katG*, codon 463).

### Assay description Luminex MOL-PCR

The assay described here is conceptually related to Multiplex Ligation-dependent Probe Amplification (MLPA) that was also described for SNP-typing of *M. tuberculosis* [25], [57]. MLPA uses allele-specific ligation and stuffer sequences of variable lengths, which allow multiplexed typing with a capillary electrophoresis device. The high level of multiplexing is made possible by the PCR-amplification of the ligated oligonucleotides rather than the amplification of template DNA. This allows the use of a universal primer-pair for the singleplex-amplification of all ligated oligonucleotides and avoids multiplex-PCR. Allele-specific ligation was recently combined with the Luminex platform and named MOL-PCR, and used for pathogen detection and SNP-typing of *Bacillus anthracis*, *Yersinia pestis*, and *Francisella tularensis* [39], [58]. MOL-PCR uses fluorescent beads and coupled *tag*-sequences instead of stuffer sequences for the multiplexed analysis. This allows the signal detection on a flow cytometer, i.e. a Luminex device. We adapted and modified MOL-PCR for typing of an 8-plex set of MTBC lineage-specific SNPs. In our assay, the *tag*-sequences complementary to *antiTag*-sequences on microspheres were included on the left-probe-oligonucleotide (LPO), i.e. preceding the 5′-end of the allele-discriminating sequence (left-hybridizing sequence, LHS), contrary to the original publication of MOL-PCR [39], where *tag*-sequences were included on the right-probe-oligonucleotide (RPO). With this modification in oligonucleotide design, two competing LPO could be used, differing only by the 3′-end (the SNP of interest) and the *tag*-sequence. This change allowed the calculation of an allelic ratio rather than a signal-to-noise ratio, and improved the sensitivity by discriminating background signal from uncalled allele signal. An additional change was made in separating the hybridization/ligation step from the PCR step. We obtained high background signal levels when combining these two steps. The assay therefore consisted of three steps (Figure 2): 1. Competitive hybridization of allele-specific probes and ligation to universal, 3′-adjacent probes. This step provides the allele-specificity of the assay. 2. Signal amplification by PCR and a fluorescently labeled primer, which guarantees the sensitivity. 3. Detection of allele-specific signals by hybridizing amplicons to allele-specific beads and read-out by flow cytometry.

### SNP selection and MOL-PCR oligonucleotide design

The selection of suitable SNPs is crucial for the success of a typing assay. In MOL-PCR, probes of 20–45 bp are designed to anneal directly adjacent to the SNP of interest. The need for a suitable sequence up- and downstream of the SNP requires a number of SNPs to choose from as a starting point. Eligible SNPs for MOL-PCR were chosen using following criteria:

1. SNP is specific for the lineage of interest
2. SNP is not in a region where a genomic deletion has been described (to ensure binding of oligonucleotides and subsequent allele-calls for all strains)
3. No other SNP less than 50 bp up- or downstream (to avoid mismatches in oligonucleotide annealing)
4. SNP is not a transition (i.e. no nucleotide change A>G, G>A, C>T or T>C; lower unspecific signal because no alternate base pairing possible)
5. SNP is in a coding sequence (deletions or insertions less likely)
6. SNP is in an essential gene (deletions less likely)
7. SNP change is synonymous (no amino acid change; therefore lower selective pressure acting on SNP compared to nonsynonymous SNP)

For each eligible SNP, 100 bp of enclosing genomic sequence of H37Rv up- and downstream of the SNP were obtained using Ugene [59] and saved as multifasta file. Sequences and SNPs were introduced in AlleleID (Premier Biosoft, Palo Alto, USA) and suitable oligonucleotides searched using the MLPA module. As MLPA uses stuffer sequences rather than *tag*-sequences (for hybridizing to microspheres with coupled *antitag*-oligonucleotides), the latter were introduced manually and according to sequences provided by Luminex Corporation (Austin, USA). We used the following criteria for oligonucleotide search in AlleleID: 1. GC-content between 35% and 65%, 2. Tm between 72°C and 90°C, 3. Minimum left and right hybridization sequence (LHS and RHS) length 21 bp. Probes with high scores in AlleleID were exported from AlleleID to OpenOffice spreadsheets. BLAST search against *M. tuberculosis* H37Rv was run to exclude cross-homology. The 24 oligonucleotides for each lineage-specific SNP were pooled (3 oligonucleotides each) and assessed for heterodimer formation with SBEprimer [60], AutoDimer [61] and Oligoanalizer (www.idtdna.com). Probes with potential dimer formation (dG<−11 kcal/mol) were excluded, a new SNP chosen and new probes designed, until a compatible set was found. The final set of 24 oligonucleotides was synthesized by Sigma-Aldrich (Buchs, Switzerland). The 5′-end of each RPO was phosphorylated to enable ligation.

### Luminex MOL-PCR procedure

All SNPs were interrogated in one tube (multiplexed). Our modified MOL-PCR assay consisted of three laboratory steps. 1. Oligonucleotides were hybridized to target DNA and ligated. 2. The ligation product was amplified by singleplex PCR (one primer pair for all SNPs). 3. Beads were hybridized to the amplification products and run on a flow cytometric device.

#### Hybridization and ligation

In a 10 µL volume with H_2_O, 0.5 µL of oligonucleotide-mix (40 nM each oligonucleotide; see Table 1) were mixed with 1 µL of ligation buffer (NEB; New England Biolabs, Ipswich, USA), 0.1 µL of thermostable ligase (NEB) and 3 µL of pure DNA (10 ng/µL) or 4 µL heat-inactivated, crude extract. In a thermocycler, the following protocol was run: 4 min 94°C, 30 cycles 25 sec 94°C and 30 sec 50°C.

**Table 1:** Oligonucleotides used for Luminex MOL-PCR.

MTBC Lineage^1^	SNP^2^	Gene	Ess/Syn^3^	Strand^4^	Allele^5^	Bead^6^	Sequence^7^
1	Rv3920109GT	yrbE4A	ess/syn	antisense	LPO_ancestral	30	GGGTTCCCTAAGGGTTGGActtaacatttaacttctataacacGTAGTCATGACCGCTATAGGTGTGCGAAT**C**
					LPO_mutant	35	GGGTTCCCTAAGGGTTGGAcatcttcatatcaattctcttattGTAGTCATGACCGCTATAGGTGTGCGAAT**A**
					RPO		P-TCGGCGCAGTAACAAACGACGGTGATCTAGATTGGATCTTGCTGGCAC
2	Rv1834177AC	rpsA	ess/syn	sense	LPO_ancestral	12	GGGTTCCCTAAGGGTTGGAcataatcaatttcaactttctactGCGAGTTCCTGAATAACTTGCAAAAAGGCACCATCCG**A**
					LPO_mutant	13	GGGTTCCCTAAGGGTTGGAcaaatacataatcttacattcactGCGAGTTCCTGAATAACTTGCAAAAAGGCACCATCCG**C**
					RPO		P-AAGGGTGTCGTGTCCTCGATCGTCAACTTCGGTCTAGATTGGATCTTGCTGGCAC
3	Rv301341CA	Rv0249c	ess/syn	sense	LPO_ancestral	18	GGGTTCCCTAAGGGTTGGAacacttatctttcaattcaattacTGAGCCGGAATCCAAGCAGGAA**C**
					LPO_mutant	19	GGGTTCCCTAAGGGTTGGAatactttacaaacaaataacacacTGAGCCGGAATCCAAGCAGGAA**A**
					RPO		P-GGTAATACCATCGCTCCCAACGGAATCTCTAGATTGGATCTTGCTGGCAC
4	Rv3326554CA	Rv2971	ess/nonsyn.	antisense	LPO_ancestral	20	GGGTTCCCTAAGGGTTGGActttctcatactttcaactaatttGACGAATGTGAGGTCGATAAGGTTTTCGATGT**G**
					LPO_mutant	21	GGGTTCCCTAAGGGTTGGAtcaaactctcaattcttacttaatGACGAATGTGAGGTCGATAAGGTTTTCGATGT**T**
					RPO		P-CTCCGCGGTGAAGTTGGACACGCCGATTCTAGATTGGATCTTGCTGGCAC
5	Rv1377185CG	Rv1234	noness/syn	sense	LPO_ancestral	36	GGGTTCCCTAAGGGTTGGAattaaacaactcttaactacacaaGCAGGTGACCATCGTTGGCGTGGACCT**C**
					LPO_mutant	37	GGGTTCCCTAAGGGTTGGAtacaacatctcattaacatatacaGCAGGTGACCATCGTTGGCGTGGACCT**G**
					RPO		P-ATGCAGGTTGAACGGGTCACAGGCCTCTAGATTGGATCTTGCTGGCAC
6	Rv2427828GC	Rv2164c	ess/syn	sense	LPO_ancestral	33	GGGTTCCCTAAGGGTTGGAactacttattctcaaactctaataCATCGGTGGACAACCACAGTGTGAG**G**
					LPO_mutant	34	GGGTTCCCTAAGGGTTGGAacttatttcttcactactatatcaCATCGGTGGACAACCACAGTGTGAG**C**
					RPO		P-CCTAGTCCGACGCCGAGCGAACCGATAATCTAGATTGGATCTTGCTGGCAC
*M. bovis*/*M. caprae*	Rv3480645TG	Rv3113	ess/nonsyn	sense	LPO_ancestral	28	GGGTTCCCTAAGGGTTGGAcacttaattcattctaaatctatcTTTCCGTCTTGGTCACCGGAGCTTCAC**T**
					LPO_mutant	29	GGGTTCCCTAAGGGTTGGAtactacttctataactcacttaaaTTTCCGTCTTGGTCACCGGAGCTTCAC**G**
					RPO		P-GCAGCCACCCGACCGAGTCATGCTCAAATCTAGATTGGATCTTGCTGGCAC
Beijing sublineage	Rv2112832AC	Rv1865c	noness/syn	sense	LPO_ancestral	14	GGGTTCCCTAAGGGTTGGAaatttcttctctttctttcacaatTTTCGCCATCGCCTCATCGATGTCGCCGAT**A**
					LPO_mutant	25	GGGTTCCCTAAGGGTTGGActttcttaatacattacaacatacTTTCGCCATCGCCTCATCGATGTCGCCGAT**C**
					RPO		P-GCGACCTTGGCTCCCAAGTTGTGCATCTAGATTGGATCTTGCTGGCAC

1 Nomenclature according to [2].

2 Position of SNP in reference to H37Rv.

3 SNP in an essential gene (“ess”); synonymous SNP (“syn”).

4 Strand orientation of oligonucleotides.

5 LPO, left probe oligonucleotide, annealing upstream of SNP of interest and including it; RPO, right probe oligonucleotide, annealing downstream of SNP of interest.

6 x*Tag* bead with coupled *antiTag*-sequence.

7 Universal primer sequences (upper case, not underlined), allele-specific *tag*-sequences (lower case), and sequences hybridizing to the template genomic sequence (underlined). RPO are 5′-phosphorylated.

*PCR:* In a 10 µL volume, 3 µL of the ligation product was added to the PCR mix of 5.095 µL of H_2_O, 1 µL PCR buffer without MgCl_2_ (Roche, Basel, Switzerland), 0.2 µL dNTPs (10 mM, Sigma-Aldrich), 0.5 µL forward primer (10 µM; Sigma-Aldrich, 5′-Alexa532-GGGTTCCCTAAGGGTTGGA), 0.125 µL reverse primer (10 µM; Sigma-Aldrich, GTGCCAGCAAGATCCAATCTAGA) and 0.08 µL FastStart Taq polymerase (Roche). The following protocol was run in a thermocycler: 2 min 96°C and 43 cycles of 20 sec 94°C, 20 sec 58°C, 20 sec 72°C.

#### Hybridization of beads and signal detection

A bead mix containing 2 µL of beads (16 polystyrene bead regions of MagPlex-Tag beads, Luminex Corp., Austin, USA; Table 1) and 3 µL of buffer (1.33 M NaCl (Merck Chemicals, Darmstadt, Germany), 83.33 mM MES (Sigma-Aldrich)) was added to the 10 µL PCR product. In a thermocycler, the following protocol was run to hybridize beads (*antiTags*) to PCR products (*tags*): 1 min 94°C, ramp-down 0.1°C/sec decrement to 25°C, 5 min 25°C. 80 µL of running buffer (10 mM Tris-HCl, 0.1 mM EDTA (Sigma-Aldrich), 90 mM NaCl, 0.04% (v/v) Triton X-100 (Sigma-Aldrich), pH 8) were added and the reaction transferred to flat-bottom plates (BioRad, Hercules, USA). A BioPlex 200 device (BioRad) was used to discriminate beads and measure reporter fluorescence (Alexa532) for each bead, i.e. each allele. Results were obtained as reporter median fluorescence intensity (MFI) per bead region and sample, and exported from BioPlex Manager software into spreadsheets.

### Allele calling MOL-PCR

For each sample, the allelic state of each SNP was assessed with the following algorithm:

1. Bead counts >40 beads per bead region
2. Allelic ratio (AR)<0.4; ancestral allele called
3. Allelic ratio (AR)>0.6; mutant allele called

AR = (MFI_mutant_/MFI_H2O_mutant_)/((MFI_mutant_/MFI_H2O_mutant_)+(MFI_ancestral_/MFI_H2O_ancestral_))

1. MFI>threshold MFI (see below)

MFI = median fluorescence intensity of reporter-dye (Alexa532) of the corresponding allele/bead region

A threshold MFI for each allele/bead was used to exclude low signal calls and to avoid SNP-calls for H_2_O signals (false-positives). This value was different for each SNP and each allele (i.e. each bead region) and had to be calculated for each run, as we observed significant differences of H_2_O signal levels between different runs, and occasionally H_2_O signal levels higher than uncalled allele signals. The pragmatic approach that we used to calculate the signal threshold for allele was the following. For each allele, the threshold was set higher than each of the corresponding H_2_O values (a minimum of three H_2_O samples per 96-well-plate were included). The threshold was then lowered so far that no allele was called for any of the H_2_O-sample, which is made possible by the range of the allelic ratio. Furthermore, an absolute minimal threshold value was used.

### Assay description TaqMan PCR

TaqMan real-time PCR was originally described in 1991 [62] and was since then optimized for commercial use. The PCR based method makes use of fluorescently labeled allele-specific probes, the 5′-3′-exonuclease activity of Taq polymerase and a non-fluorescent quencher. During PCR amplification of the region of interest, fluorescence is detected in a real-time PCR thermocycler.

### SNP selection and probe design for TaqMan

Primers and probes for Lineage 2, Lineage 3 and Lineage 4 were described before (Table 2). SNP for Lineage 1 was chosen from the list of lineage-specific SNPs recently published [10], and SNPs for Lineage 5 and 6 from the list of genome sequences as described above. For the design of probes and primers, the software Primer Express (Applied Biosystems, Carlsbad, USA) was used. As TaqMan assays were singleplex, oligonucleotide design was less complex, as heterodimer formation is limited to the two primers and two probes of one assay. Furthermore, the design of the primer pair can be varied over a large sequence range.

**Table 2:** Primer and probe sequences for TaqMan assays.

MTBC Lineage^1^	LSP name^2^	Spoligotype name^3^	SNP^4^	Forward primer	Reverse primer	Ancestral allele probe^5^	Mutant allele probe^5^	Reference
1	Indo-oceanic	EAI, MANU1	Rv3597682CT	TGTCAACGAAGGCGATCAGA	GACCGTTCCGGCAGCTT	6FAM-ACAAGGGCGACGTC-MGBNFQ	VIC-ACAAGGGCGACATC-MGBNFQ	This study
2	East Asian	Beijing	Rv3304966GA	CCTTCGATGTTGTGCTCAATGT	CATGCGGCGATCTCATTGT	6FAM-CCCAGGAGGGTAC-MGBNFQ	VIC-CCCAGGAAGGTACT- MGBNFQ	[14]
3	East-African-Indian	CAS	Rv4266647AG	GCATGGATGCGTTGAGATGA	CGAGTCGACGCGACATACC	VIC-AAGAATGCAGCTTGTTGA-MGBNFQ	6FAM-AAGAATGCAGCTTGTCGA- MGBNFQ	[14]
4	Euro-American	X, Haarlem, LAM, Uganda	Rv2154724AC	CCGAGATTGCCAGCCTTAAG	GAAACTAGCTGTGAGACAGTCAATCC	VIC-CCAGATCCTGGCATC-MGBNFQ	6FAM-CAGATCCGGGCATC- MGBNFQ	[20]
5	*M. africanum* West African I	AFRI 2	Rv1377185GC	TCCAGCAGGTGACCATCGT	GGCCTGTGACCCGTTCAAC	VIC-CGTGGACCTCATG-MGBNFQ	6FAM-CGTGGACCTGATGC- MGBNFQ	This study
6	*M. africanum* West African II	AFRI 1	Rv378404GA	CGGCCGACAGCGAGAA	CCATCACGACCGAATGCTT	6FAM-CTGCAAATCCCGCAGTA-MGBNFQ	VIC-CTGCAAATCCCACAGT- MGBNFQ	This study

1 Nomenclature according to [2].

2 Nomenclature according to [20].

3 Nomenclature according to [72].

4 Position of SNP in reference to the H37Rv genome.

5 6FAM and VIC, fluorescent dyes at the 5′-end of probes; MGBNFQ, MinorGrooveBinder-NonFluorescentQuencher at the 3′-end.

### TaqMan real-time PCR

TaqMan real-time PCR was performed according to standard protocols (Applied Biosystems, Carlsbad, USA). Briefly, in a 12 µL volume, 2 µL DNA sample were added to a mix of 5 µL TaqMan Universal MasterMix II (Applied Biosystems), 5 µL H_2_O containing forward and reverse primer (0.21 µM each; Sigma-Aldrich), probe A for ancestral allele and probe B for mutant allele (0.83 µM each; Applied Biosystems). Primer and probe sequences are listed in Table 2. Reactions were run in a StepOnePlus thermocycler (Applied Biosystems; 60°C 30 sec; 95°C 10 min; 95°C 15 sec and 60°C 1 min for 40 cycles; 60°C 30 sec) and fluorescence intensity in the VIC and FAM channels measured at the end of every cycle. Results were analyzed with StepOne software (Applied Biosystems) and alleles called with the default algorithm.

### Spoligotyping

43-spacer spoligotyping was performed following standard protocols on a membrane [12].

### PCR and sequencing of putative deletion in Rv3113

As we did not obtain MOL-PCR signal for the *M. bovis*/*M. caprae*-specific SNP for two strains (N1007 and N1032, see Table S1), the locus was assessed for a potential deletion in Rv3113. PCR was run in a 25 µL volume with 12.3 µL H_2_O, 2.5 µL PCR buffer with MgCl2 (Roche), 5 µL GC buffer, 0.75 µL forward primer K-297 (10 µM, Sigma-Aldrich, CCATGATGCTGGCAGAACTGA), 0.75 µL reverse primer K-298 (10 µM, Sigma-Aldrich, CCTGCGTACCTTCGTCGTCA), 0.5 µL dNTPs (10 mM, Qiagen, Hombrechtikon, Switzerland) and 0.2 µL Fast Start Taq Polymerase (Roche). Reaction was run in a standard thermocycler (96°C, 7 min; 35 cycles of 96°C 30 sec, 62°C 30 sec, 72°C 30 sec; 72°C 4 min). PCR product was analyzed using standard gel electrophoresis and stained with ethidium bromide.

## Results

Eight SNPs were used in the multiplexed MOL-PCR assay (Table 1). Six SNPs were interrogated serially with TaqMan assays (Table 2). Forty-six MTBC strains were run as a “training panel” (Table S1). Most samples were available as 10 ng/µL purified genomic DNA (CTAB-method) and as crude lysates from 7H9 culture. Both methods, MOL-PCR and TaqMan, were run in parallel for all samples and all SNPs. Table S1 shows allelic state for each SNP for both purified DNAs and crude extracts, called by MOL-PCR and TaqMan, respectively.

For MOL-PCR, 662 of 672 (98.6%) total data points (alleles) resulted in a successful call of either the ancestral or the mutant allele (Table S1 and Table 3). Eight data points, for which no allele call was obtained, involved crude lysates, and two were purified DNA samples. For two strains, N1007 and N1032, no allele was called for the *M. bovis*/*M. caprae*-specific SNP, Rv3480645TG, for both crude and purified DNA. To test for a potential deletion in the corresponding genomic region, we designed PCR primers and amplified the region up- and downstream of this SNP. Gel electrophoresis showed an expected amplicon length of approximately 250 bp for H37Rv, but no band for the two strains in question, suggesting that this region was deleted in strains N1007 and N1032 (Figure S1).

**Table 3:** Comparison of MOL-PCR and TaqMan for allele calling and lineage assignment.

		MOL-PCR	TaqMan
Training panel	Total data points	672 (100%)^1^	504 (100%)^1^
	Data points with allele call	662 (98.6%)	498 (98.8%)
	Total number of strains	42 (100%)^2^	36 (100%)^3^
	Strains assigned to a lineage	42 (100%)	36 (100%)
	Congruence in lineage assignment	100%	
Test panel	Total data points	624 (100%)	78 (100%)
	Data points with allele call	619 (99.2%)	78 (100%)
	Total number of strains	78	78
	Strains assigned to a lineage	76 (97.4%)	78 (100%)
	Congruence in lineage assignment	100%	

1 excluding N/A as indicated in Table S1.

2 excluding M. microti, M. pinnipedi.

3 excluding *M. bovis*, *M. caprae*, *M. microti*, *M. pinnipedii*.

For TaqMan PCR, 498 alleles of 504 (98.8%) total data points were successfully called (Table S1 and Table 3). The remaining six data points without allele calls were two crude lysates and four purified DNA samples. There was no case where TaqMan PCR failed to call an allele for both purified and heat-inactivated sample.

Both methods agreed 100% in lineage assignment of the 36 strains comprising the training panel (excluding *M. bovis*, *M. caprae*, *M. microti* and *M. pinnipedi*, as no SNP for *M. bovis*/*M. caprae* was used in the TaqMan assay) (Table 4). Using the prototype spacer definitions [63], [64], spoligotyping data confirmed the lineage-assignments of TaqMan and MOL-PCR for 39 of 42 strains (excluding *M. microti*/*M. pinnipedii*). Of the three remaining strains, N0153 and N0051 could not be classified into any of the known spoligotype families. Strain N1024 showed a “Beijing” spoligopattern and was confirmed by TaqMan and MOL-PCR as a Lineage 3 (also known as CAS/Dehli) strain (Table 3). The spoligotype of this strain was described as “Pseudo-Beijing” previously [14].

**Table 4:** Lineage assignments of MOL-PCR, TaqMan and spoligotyping for the 46 MTBC strains as in Table S1.

	Lineage assignment					
MTBC strain	MOL-PCR	TaqMan	Spoligotyping^1^	Spoligotype	SITVIT lineage^2^	SIT^2^
N1004	1	1	1	▪▪▪▪▪▪▪▪▪▪▪▪▪▪▪▪▪▪▪▪▪▪□▪▪▪▪▪□□□□▪□▪▪□▪▪▪▪▪▪	EAI6-BGD1	292
N1006	1	1	1	▪▪▪▪▪▪▪▪▪▪▪▪▪▪▪▪▪▪▪▪▪▪▪▪▪▪▪▪□□□□▪□□□▪▪▪□□□□	EAI5	ORPHAN
N0153	1	1	-3	▪▪▪▪▪▪▪▪▪▪▪▪▪▪▪▪▪▪□□□□□□□□□□□□□□□□□□□□□□□▪▪	ZERO	405
N1011	1	1	1	▪▪▪▪▪▪▪▪▪▪▪▪▪▪▪▪▪▪▪▪▪▪▪▪▪▪▪▪□□□□▪□□□▪▪▪□□□□	EAI5	ORPHAN
N1030	1	1	1	▪▪▪▪▪▪▪▪▪▪▪▪▪▪▪▪▪▪▪▪▪▪▪▪▪▪▪▪□□□□▪□▪▪▪▪▪□□□□	EAI5	138
N1068	1	1	1	▪□□▪▪▪▪▪▪▪▪▪▪□▪▪▪▪▪▪▪▪▪▪▪▪▪▪□□□□▪□▪▪□□□▪▪▪▪	EAI3-IND	ORPHAN
N1053	2/Beijing	2	2	□□□□□□□□□□□□□□□□□□□□□□□□□□□□□□□□□□▪▪▪▪▪▪▪▪▪	BEIJING	1
N1002	2/Beijing	2	2	□□□□□□□□□□□□□□□□□□□□□□□□□□□□□□□□□□▪▪▪▪▪▪▪▪▪	BEIJING	1
N1010	2/Beijing	2	2	□□□□□□□□□□□□□□□□□□□□□□□□□□□□□□□□□□▪▪▪▪▪▪▪▪▪	BEIJING	1
N1012	2/Beijing	2	2	□□□□□□□□□□□□□□□□□□□□□□□□□□□□□□□□□□▪▪▪▪▪▪▪▪▪	BEIJING	1
N0051	2/non-Beijing	2	-3	□□□□□□□□□□□□□□□□□□□□□□□□▪▪▪▪▪▪▪▪▪▪▪▪▪▪▪▪▪▪▪	-	955
N1069	2/Beijing	2	2	□□□□□□□□□□□□□□□□□□□□□□□□□□□□□□□□□□▪▪▪▪▪▪▪▪▪	BEIJING	1
N1013	2/Beijing	2	2	□□□□□□□□□□□□□□□□□□□□□□□□□□□□□□□□□□▪▪▪▪▪▪▪▪▪	BEIJING	1
N1144	3	3	3	▪▪▪□□□□▪▪▪▪▪▪▪▪▪▪▪▪▪▪▪□□□□□□□□□□□□▪▪▪▪▪▪▪▪▪	CAS1-DELHI	26
N1007	3	3	3	▪▪▪□□□□▪▪▪▪▪▪▪▪▪▪▪▪▪▪□□□□□□□□□□□□□▪▪▪▪▪▪▪▪▪	CAS	142
N1082	3	3	3	▪▪▪□□□□▪▪▪▪▪▪▪▪▪□▪▪▪▪▪□□□□□□□□□□□□▪▪▪□□□□□▪	-	-
N1024	3	3	-4	□□□□□□□□□□□□□□□□□□□□□□□□□□□□□□□□□□▪▪▪▪▪▪▪▪▪	BEIJING	1
N1032	3	3	3	▪▪▪□□□□▪▪▪▪▪▪▪▪▪▪▪▪▪▪▪□□□□□□□□□□□□▪▪▪▪▪▪▪▪▪	CAS1-DELHI	26
N1021	3	3	3	▪▪▪□□□□▪▪▪▪▪▪▪▪▪▪▪▪▪▪▪□□□□□□□□□□□□□□▪▪▪▪▪▪▪	CAS	357
N1022	3	3	3	▪▪□□□□□▪▪▪▪▪▪▪▪▪▪▪▪▪▪▪□□□□□□□□□□□□▪▪□□▪▪▪▪▪	-	2485
H37Rv	4	4	4	▪▪▪▪▪▪▪▪▪▪▪▪▪▪▪▪▪▪▪□□▪▪▪▪▪▪▪▪▪▪▪□□□□▪▪▪▪▪▪▪	H37RV	451
N1008	4	4	4	▪□□▪▪▪▪▪▪▪▪▪▪▪▪▪▪▪▪▪▪▪▪▪▪▪▪▪▪▪□▪□□□□▪▪▪▪▪▪▪	H3	655
N1015	4	4	4	▪▪▪▪▪▪▪▪▪▪▪▪□□□□▪▪▪▪▪▪▪▪▪▪▪▪▪▪▪▪□□□□▪▪▪▪▪▪▪	T1	102
N1019	4	4	4	▪▪▪□▪▪▪▪□□▪▪□▪▪▪▪▪▪▪▪▪▪▪▪▪▪▪▪▪▪▪□□□□▪▪▪▪□▪▪	-	-
N1052	4	4	4	▪▪▪▪▪▪▪▪▪▪▪▪▪▪▪▪▪▪▪▪▪▪▪▪▪▪▪▪▪▪▪▪□□□□▪▪▪▪▪▪▪	T1	53
N1057	4	4	4	▪▪▪▪▪▪▪▪▪▪▪▪▪▪▪▪▪▪▪▪▪▪▪▪▪▪▪▪▪▪▪▪□□□□▪▪□□□□▪	T1	244
N1034	5	5	5	▪▪▪▪▪▪▪□□□□□▪▪▪▪▪▪▪▪▪▪▪▪□▪□□□□□□□□□▪□□□▪▪▪▪	-	-
N1035	5	5	5	▪▪▪▪▪▪▪□□□□□▪▪▪▪▪▪▪▪▪▪▪▪□▪□□□□□□□□□▪□□□□▪▪▪	-	ORPHAN
N1063	5	5	5	▪▪▪▪▪▪▪□□□□□□▪▪▪▪▪▪▪□□□□▪▪▪▪▪▪▪□□□□□□□□▪▪▪▪	-	-
N1064	5	5	5	▪▪▪▪▪▪▪□□□□□▪▪▪▪▪▪▪▪□□□□▪▪□▪▪▪▪▪□▪▪▪□□□▪▪▪▪	AFRI_2	101
N1286	5	5	5	▪▪▪▪▪▪▪□□□□□▪▪▪▪▪▪▪□□□□□□□□▪▪□□□□□▪▪□□□□▪▪▪	-	-
N1201	6	6	6	▪▪▪▪▪▪□□□▪▪▪▪▪▪▪▪▪▪▪▪□□□▪▪▪▪▪▪▪▪▪▪▪▪▪▪□□▪▪▪	-	3476
N1292	6	6	6	▪▪▪▪▪▪□□□▪□□▪▪▪▪▪▪▪▪▪□□□▪▪▪▪▪▪▪▪▪▪▪▪▪▪□▪▪▪▪	-	-
N0091	6	6	6	▪▪▪▪▪▪□□□▪▪▪▪▪▪▪▪▪▪▪▪▪▪▪▪▪▪▪▪▪▪▪▪▪▪▪▪▪□▪▪▪▪	AFRI_1	181
N0092	6	6	6	▪▪▪▪▪▪□□□▪▪▪▪▪▪▪▪▪▪▪▪▪▪▪▪▪▪▪▪▪▪▪▪▪▪▪▪▪□▪▪▪▪	AFRI_1	181
N0115	6	6	6	▪▪▪▪▪▪□□□▪▪▪▪▪▪▪▪▪▪▪▪▪▪▪▪▪▪▪▪▪▪▪▪▪▪▪▪▪□▪▪▪▪	AFRI_1	181
*M. bovis* BCG Pasteur	*M. bovis*/*M. caprae*	(N/A)	*M. bovis*	▪▪□▪▪▪▪▪□▪▪▪▪▪▪□▪▪▪▪▪▪▪▪▪▪▪▪▪▪▪▪▪▪▪▪▪▪□□□□□	BOV_1	482
*M. bovis* BCG Montreal	*M. bovis*/*M. caprae*	(N/A)	*M. bovis*	▪▪□▪▪▪▪▪□▪▪▪▪▪▪□▪▪▪▪▪▪▪▪▪▪▪▪▪▪▪▪▪▪▪▪▪▪□□□□□	BOV_1	482
*M. bovis* 1290-03	*M. bovis*/*M. caprae*	(N/A)	*M. bovis*	▪▪□□□▪▪▪□▪▪▪▪▪▪□▪▪▪▪▪▪▪▪▪▪▪▪▪▪▪▪▪▪▪▪▪▪□□□□□	BOV_1	665
*M. bovis* 8217-02	*M. bovis*/*M. caprae*	(N/A)	*M. bovis*	▪▪□▪▪▪▪▪□▪▪▪▪▪▪□▪▪▪▪▪▪▪▪▪▪▪▪▪□▪▪▪▪▪▪▪▪□□□□□	BOV_1	1037
*M. caprae* 7140-99	*M. bovis*/*M. caprae*	(N/A)	(N/A)	□▪□□□□□□□□□□□□□□▪▪▪▪▪▪▪▪▪▪▪□▪▪▪▪▪▪▪▪▪▪□□□□□	BOV4_CAPRAE	647
*M. caprae* 8986-99	*M. bovis*/*M. caprae*	(N/A)	(N/A)	□▪□□□□□□□□□□□□□□▪▪▪▪▪▪▪▪□□□□□□□□□□□□□□□□□□□	BOV4_CAPRAE	818
*M. microti* 1479-00	-	(N/A)	(N/A)	□□□□□□□□□□□□□□□□□□□□□□□□□□□□□□□□□□□□▪▪□□□□□	MICROTI	539
*M. microti* 8753-00	-	(N/A)	(N/A)	□□□▪▪▪▪□□□□□□□□□□□□□□□▪▪□▪□□□□□□□□□□▪▪□□□□□	PINI1	642
*M. pinnipedii* 7011-02	-	(N/A)	(N/A)	□□□▪▪▪▪□□□□□□□□□□□□□□□▪▪▪▪□□▪▪▪▪▪▪▪▪▪▪□□□□□	-	-
*M. pinnipedii* 7739-01	-	(N/A)	(N/A)	□□□▪▪▪▪□□□□□□□□□□□□□□□▪▪▪▪□□▪▪▪▪▪▪▪▪▪▪□□□□□	-	-

1 Lineage-classification using spoligotype prototypes as described in [10], [63], [64].

2 As described in [64].

3 No lineage-classification possible based on spoligotyping.

4 “Pseudo-Beijing” as described in [14].

Next, we used a test panel of 78 clinical isolates from Nepal to validate our methods (Table S2). These samples were mainly crude, heat-inactivated extracts, and no genotyping had been done before. To follow the procedure that would be used as routine SNP-typing, we first performed MOL-PCR, as information for all SNPs is obtained in parallel. Of a total of 624 data points (i.e. 78 strains and eight SNPs), 619 (99.2%) allele calls were obtained (Table S2 and Table 3). The five data points with no calls were from three strains (1671A, 3052B and 3074B). Thereof, two strains (1671A, 3074B) failed to be assigned to a lineage by MOL-PCR (97.4% sensitivity in lineage assignment). Nevertheless, we obtained ancestral allele calls for seven (1671B) and six (3074B) other SNPs for these strains. We then performed the corresponding TaqMan assay for each sample. TaqMan called alleles for all 78 samples. Again, lineage-assignment was 100% congruent between MOL-PCR and TaqMan.

To evaluate the signal detection limit of MOL-PCR, we used different DNA concentrations as input material. Samples with known DNA concentration were diluted appropriately for different amounts of input DNA. One strain of each lineage was used and MOL-PCR performed with 60 ng, 10 ng, 1 ng, 0.1 ng purified DNA, and 4 µL of crude, heat-inactivated extract. Figures S2, S3, S4, S5, S6, S7 and S8 show a decrease of signal intensities with decreasing amount of DNA. Nevertheless, the use of 0.1 ng purified DNA (CTAB) or 4 µL crude extract still resulted in signals high enough for allelic ratio determination. Signal intensities of the complementary allele were constantly low.

## Discussion

Informative SNPs are valuable markers for deep phylogenetic typing of clinical MTBC isolates [11]. Therefore, SNP-typing will likely play an important role for genotype-phenotype association studies in the coming years. Even though routine whole-genome-sequencing, i.e. “genometyping”, might replace most other typing methods eventually, current limitations such as costs, bioinformatics capacity, the lack of user-friendly software packages, and data storage has been hindering the broad implementation of “genometyping” [1], [11]. Nevertheless, thousands of informative SNPs have become available from whole genome sequences, which can be used for SNP-typing.

In this study, we presented 14 canonical SNPs, comprising two sets of markers for the six main phylogenetic lineages of MTBC, the *M. bovis*/*M. caprae* clade and the “Beijing” sublineage. In contrast to other published SNP sets [23], [24], our SNPs were obtained from many whole genome sequences representing the global diversity of MTBC, and thus do not suffer from phylogenetic discovery bias [65]. Phylogenetic discovery bias occurs when SNPs used for subsequent genotyping are initially identified by comparing only a few whole genome sequences (for example when these genomes represent only a subset of all MTBC lineages). As a result, strains belonging to lineages not represented in the initial genome set will falsely appear as intermediates in the corresponding phylogenies [66]. In additional advantage of our SNP sets is that they also identify *Mycobacterium africanum* [67] and discriminate between the West-African I and the West-African II subtypes (i.e. MTBC Lineages 5 and 6). Moreover, the addition of a “Beijing”-specific SNP makes the differentiation between this sublineage and other strains of Lineage 2 (East-Asian) possible.

For the rapid typing of the SNPs described, we have successfully developed a multiplexed assay adapted from MOL-PCR [39]. Our assay uses three oligonucleotides per SNP instead of two, with the LPOs competing for annealing to the template DNA. This allows an allele-call based on allelic ratio rather than signal-to-noise ratio, which increases sensitivity. We further separated the hybridization/ligation from the PCR step, which reduced unspecific signal. We compared the new MOL-PCR assay with TaqMan real-time PCR which was used as standard SNP-typing method in our laboratory. We found an agreement of 100% in classifying strains into the main phylogenetic lineages and an allele-calling sensitivity of 98.6% and 98.8%, respectively, when typing two panels of clinical MTBC strains.

MOL-PCR was sensitive enough to be used with low concentrations of DNA as well as heat-inactivated samples (crude extracts) (Figures S2, S3, S4, S5, S6, S7 and S8). We found that the detection limit of MOL-PCR was as low as 0.1 ng of input DNA. Heat-inactivated samples resulted in similarly high signal levels. This is especially valuable as performing MTBC cultures and DNA extraction is laborious and time-consuming. Even though the signal levels of H_2_O control reactions were at time relatively high (Figures S2, S3, S4, S5, S6, S7 and S8), the MFI threshold determination we applied allowed us to control for this, and water samples could not be called false-positive alleles. We recommend to include at least three water samples per 96-well plate, and to use a mean value for the calculation of allelic ratios. Additionally, we always included H_2_O control samples for the PCR step to detect potential contamination.

We observed a putative deletion in the region of SNP Rv3480645TG, as we did not obtain signal for neither allele for two samples (N1007 and N1032). To our knowledge, no genomic deletion has been described to span this SNP [19]. PCR amplification of this region resulted in no visible band for these two samples (Figure S1), which suggested that there might be a deletion present in some Lineage 3 (CAS/Delhi) strains. This highlights the fact that genomic deletions can affect a SNP call, even though the corresponding gene was originally described as essential [54], [55]. However, with MOL-PCR we always generated complete allelic information for all other SNPs of the assay. As the canonical SNPs defining different MTBC lineages are mutually exclusive (except Lineage 2 and Beijing), the lack of an allelic call for one SNP can be neglected if the alleles at other loci are called successfully.

A limitation in the development of the MOL-PCR assay was the low efficiency of design and validation of oligonucleotides. For each lineage, we had to design up to 5 sets of probes targeting different SNPs, because many had to be rejected during validation, most often because of unfavorable allelic or signal-to-noise ratios or lack of signal. The use of long (i.e. 40–140 bp) oligonucleotides makes hairpin and homodimer formations likely, and multiplexing probes adds a level of complexity due to a high combinatorial number of possible heterodimer formations. We used different software for homo- and heterodimer analysis, but still were not able to fully predict the success of probes. Recently, an online tool for MOL-PCR specific oligonucleotide design and evaluation became available (moligodesigner.lanl.gov) [58], which could strongly facilitate the design of such oligonucleotides. Furthermore, an oligonucleotide design tool for copy-number-variation analysis of human, mouse and rat genomes with MLPA and MOL-PCR has been described [68], [69], and might be extended to SNP analysis and bacterial genomes.

Four other methods have been described for multiplexed SNP-typing on the Luminex platform, namely Direct Hybridization, Allele-Specific Primer Extension, Single-Base-Extension and Oligonucleotide Ligation [40], [41]. In contrast to MOL-PCR, these methods use multiplex-PCR for amplification of template DNA. This makes high multiplex-levels difficult, as a multiplex PCR is usually the limiting factor in multiplexed assays. In our assay, 24 oligonucleotides of 48 to 80 bp lengths were included for eight SNPs. A number of additional SNPs could be interrogated in the same reaction, i.e. for sublineages, drug resistance mutations [25] or outbreak-specific SNPs [70].

In addition to MOL-PCR, we also developed TaqMan SNP-assays for robust and rapid singleplex-typing of all main MTBC lineages. TaqMan SNP-typing is a well-established method for genotyping and available commercially. This facilitates the design and validation of probes, and provides standardized reaction conditions. Furthermore, criteria of TaqMan primer and probe design are less strict than in MOL-PCR. TaqMan makes use of quantitative PCR, which renders the method particularly sensitive. On the other hand, multiplexing of TaqMan assays is demanding and the degree of multiplexing is limited. Hence, we recommend using MOL-PCR for strain collections with no *a priori* information. TaqMan SNP-typing, on the other hand, is most suitable for confirmation of MOL-PCR results, or for strain collections for which previous information about genotypes is available.

Costs for reagents and consumables are another important consideration when implementing a new technique. Similar to other methods, running costs of MOL-PCR per SNP decrease with increasing number of multiplexed SNPs. We have estimated the minimal reagent costs per SNP to be ∼0.8 Euro when run in a singleplex reaction. Reagent costs drop to ∼0.15 Euro per SNP when running an 8-plex reaction. The cost of one TaqMan reaction was estimated at ∼0.9 Euro per SNP. For both assays, the initial setup costs are relatively high due to purchase of customized and labeled oligonucleotides. Furthermore, both methods require an expensive piece of equipment in form of a Luminex or a real-time-PCR machine. However, both of these devices can be used for a wide variety of applications in addition to SNP-typing (e.g. spoligotyping on the Luminex platform [71]), and many laboratories already have access to such equipment.

In conclusion, we propose a new set of canonical SNPs specific for the main phylogenetic lineages of MTBC. When combined with multiplexed MOL-PCR or singleplex TaqMan SNP-typing, these SNPs are ideal for phylotyping of strain collections from a small to a large scale. These assays provide a new basis for phylogenetically robust classification of clinical isolates.

## Supporting Information

Figure S1 **Agarose gel of PCR product of Rv3114 covering the SNP specific for** ***M. bovis*** **/** ***M. caprae*** **(Rv3480645TG).** H37Rv was used as positive control, whereas N1007 and N1032 were the samples that did not result in any signal in MOL-PCR. Ladder is Hyperladder II (Bioline).
(PDF)

Figure S2 **Dilution series of DNA from a clinical isolate of Lineage 1.** Strain N1004 was previously characterized as strain of Lineage 1. Median reporter fluorescence intensities (Alexa532-primer) of MOL-PCR are shown for each bead region and by different DNA amounts (CTAB-extracted) or crude extract (heat-inactivated). Error bars represent standard deviations calculated from triplicates.
(PDF)

Figure S3 **Dilution series of DNA from a clinical isolate of Lineage 2/Beijing.** Strain N1053 was previously characterized as strain of Lineage 2/Beijing. Median reporter fluorescence intensities (Alexa532-primer) of MOL-PCR are shown for each bead region and by different DNA amounts (CTAB-extracted) or crude extract (heat-inactivated). Error bars represent standard deviations calculated from triplicates.
(PDF)

Figure S4 **Dilution series of DNA from a clinical isolate of Lineage 3.** Strain N1144 was previously characterized as strain of Lineage 3. Median reporter fluorescence intensities (Alexa532-primer) of MOL-PCR are shown for each bead region and by different DNA amounts (CTAB-extracted) or crude extract (heat-inactivated). Error bars represent standard deviations calculated from triplicates.
(PDF)

Figure S5 **Dilution series of DNA from a clinical isolate of Lineage 4.** Strain N1015 was previously characterized as strain of Lineage 4. Median reporter fluorescence intensities (Alexa532-primer) of MOL-PCR are shown for each bead region and by different DNA amounts (CTAB-extracted) or crude extract (heat-inactivated). Error bars represent standard deviations calculated from triplicates.
(PDF)

Figure S6 **Dilution series of DNA from a clinical isolate of Lineage 5.** Strain N1286 was previously characterized as strain of Lineage 5. Median reporter fluorescence intensities (Alexa532-primer) of MOL-PCR are shown for each bead region and by different DNA amounts (CTAB-extracted) or crude extract (heat-inactivated). Error bars represent standard deviations calculated from triplicates.
(PDF)

Figure S7 **Dilution series of DNA from a clinical isolate of Lineage 6.** Strain N1200 was previously characterized as strain of Lineage 6. Median reporter fluorescence intensities (Alexa532-primer) of MOL-PCR are shown for each bead region and by different DNA amounts (CTAB-extracted) or crude extract (heat-inactivated). Error bars represent standard deviations calculated from triplicates.
(PDF)

Figure S8 **Dilution series of DNA from** ***M. bovis*** **BCG Pasteur.** Median reporter fluorescence intensities (Alexa532-primer) of MOL-PCR are shown for each bead region and by different DNA amounts (CTAB-extracted) or crude extract (heat-inactivated). Error bars represent standard deviations calculated from triplicates.
(PDF)

Table S1 **MOL-PCR and TaqMan allele calls for the training panel of 46 well-characterized MTBC strains.**
(XLS)

Table S2 **MOL-PCR and TaqMan allele calls for the test panel of 78 MTBC strains with no previous genotyping information.**
(XLS)
