# Supplementary material for: Two New Rapid SNP-Typing Methods for Classifying Mycobacterium tuberculosis Complex into the Main Phylogenetic Lineages
Source: PLoS One. 2012 Jul 20;7(7):e41253. doi: 10.1371/journal.pone.0041253 (PMC3401130; doi:10.1371/journal.pone.0041253)
Supplement: Figure S1 — Agarose gel of PCR product of Rv3114 covering the SNP specific for M. bovis / M. caprae (Rv3480645TG). H37Rv was used as positive control, whereas N1007 and N1032 were the samples that did not result in any signal in MOL-PCR. Ladder is Hyperladder II (Bioline). (PDF) [file pone.0041253.s001.pdf]

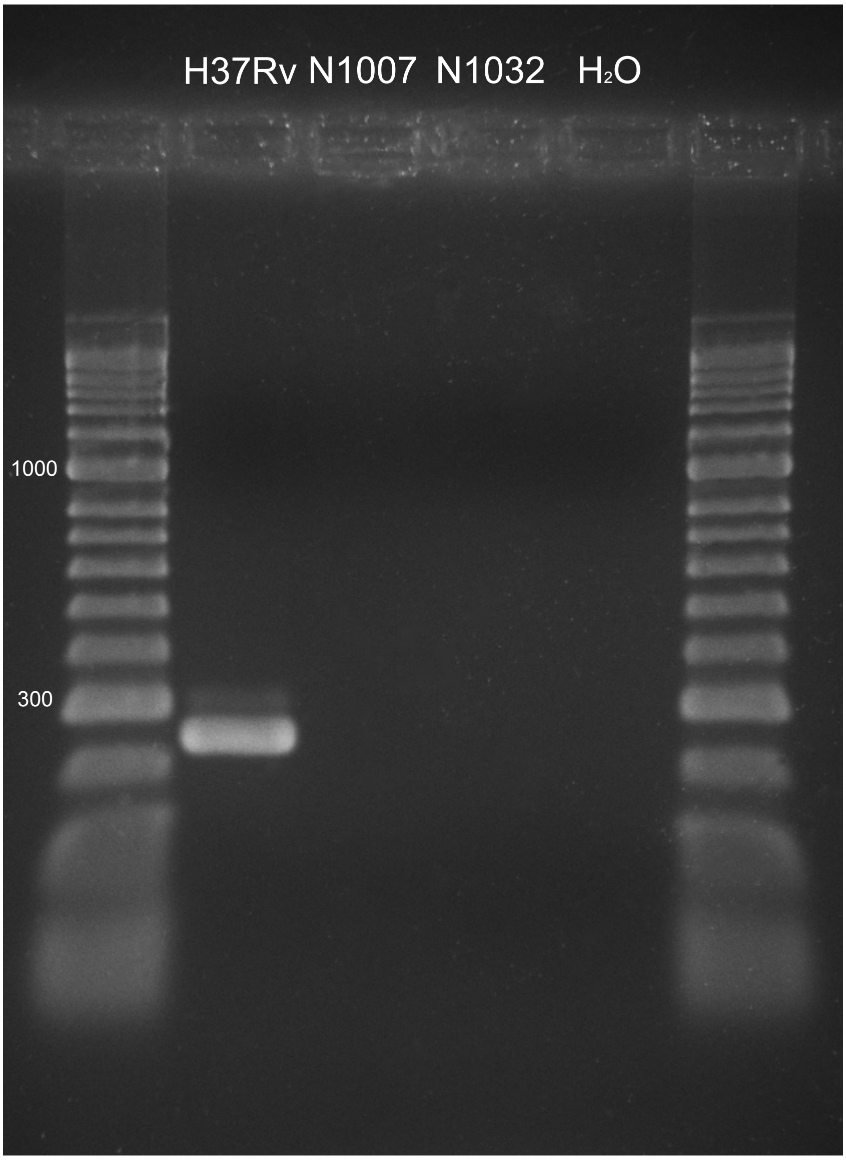

Figure S1. PCR of Rv3114 covering the SNP specific for the *M. bovis* / *M. caprae* clade (Rv3480645TG). H37Rv was used as positive control (signal in MOL-PCR obtained), whereas N1007 and N1032 were the samples that did not result in any signal in MOL-PCR. Ladder is Hyperladder II (Bioline).
