# Supplementary material for: Two New Rapid SNP-Typing Methods for Classifying Mycobacterium tuberculosis Complex into the Main Phylogenetic Lineages
Source: PLoS One. 2012 Jul 20;7(7):e41253. doi: 10.1371/journal.pone.0041253 (PMC3401130; doi:10.1371/journal.pone.0041253)
Supplement: Figure S7 — Dilution series of DNA from a clinical isolate of Lineage 6. Strain N1200 was previously characterized as strain of Lineage 6. Median reporter fluorescence intensities (Alexa532-primer) of MOL-PCR are shown for each bead region and by different DNA amounts (CTAB-extracted) or crude extract (heat-inactivated). Error bars represent standard deviations calculated from triplicates. (PDF) [file pone.0041253.s007.pdf]

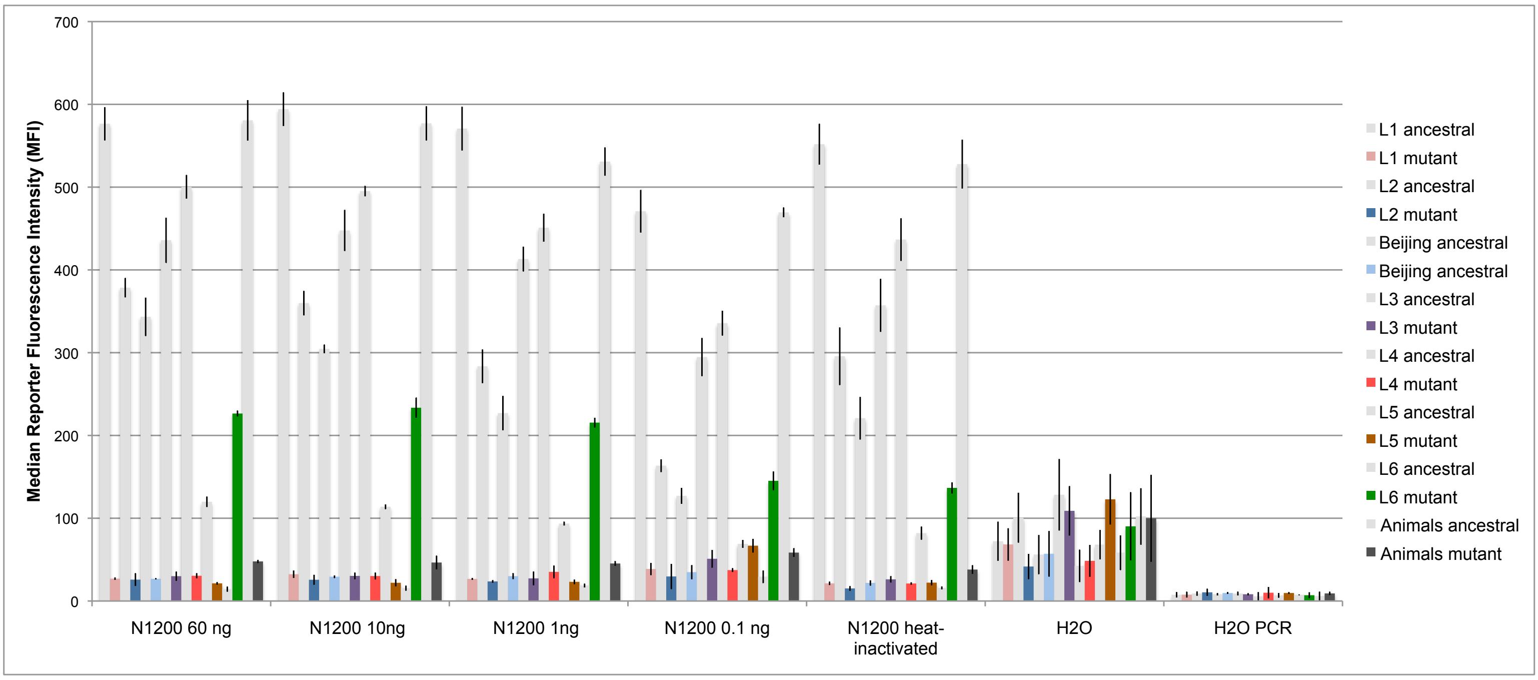

Figure S7. Dilution series of DNA from a clinical isolate of Lineage 6.
Strain N1200 was previously characterized as strain of Lineage 6. Median reporter fluorescence intensity (Alexa532-primer) of MOL-PCR shown for each bead region and by different DNA amounts (CTAB-extracted) or crude extract (heat-inactivated). Error bars represent standard deviations calculated from triplicates.
